# Activation of β-adrenergic receptors is required for elevated α1A-adrenoreceptors expression and signaling in mesenchymal stromal cells

**DOI:** 10.1038/srep32835

**Published:** 2016-09-06

**Authors:** Pyotr A. Tyurin-Kuzmin, Julia I. Fadeeva, Margarita A. Kanareikina, Natalia I. Kalinina, Veronika Yu. Sysoeva, Daniyar T. Dyikanov, Dmitriy V. Stambolsky, Vsevolod A. Tkachuk

**Affiliations:** 1 Department of Biochemistry and Molecular Medicine, Faculty of Fundamental Medicine, M.V. Lomonosov Moscow State University, Moscow, Russia

## Abstract

Sympathetic neurons are important components of mesenchymal stem cells (MSCs) niche and noradrenaline regulates biological activities of these cells. Here we examined the mechanisms of regulation of MSCs responsiveness to noradrenaline. Using flow cytometry, we demonstrated that α1A adrenergic receptors isoform was the most abundant in adipose tissue-derived MSCs. Using calcium imaging in single cells, we demonstrated that only 6.9 ± 0.8% of MSCs responded to noradrenaline by intracellular calcium release. Noradrenaline increases MSCs sensitivity to catecholamines in a transitory mode. Within 6 hrs after incubation with noradrenaline the proportion of cells responding by Ca^2+^ release to the fresh noradrenaline addition has doubled but declined to the baseline after 24 hrs. Increased sensitivity was due to the elevated quantities of α1A-adrenergic receptors on MSCs. Such elevation depended on the stimulation of β-adrenergic receptors and adenylate cyclase activation. The data for the first time clarify mechanisms of regulation of MSCs sensitivity to noradrenaline.

Mesenchymal stem cells (MSCs) were identified in the stromal-vascular compartment within the most of adult tissues including bone marrow, fat and skeletal muscles^1^. MSCs mediate physiological replenishment of connective tissues by differentiation into multiple directions such as bone, fat and cartilage. They can also enhance tissue regeneration by producing a wide range of bioactive molecules, including growth factors and cytokines, such as, vascular endothelial growth factor (VEGF), hepatocyte growth factor (HGF), insulin growth factor-1 (IGF-1) and angiopoietin-1^2^,^3^,^4^. Recently sympathetic neurons were identified as important component of MSCs niche^5^. Noradrenaline produced by such neurons can inhibit adipogenic and osteogenic differentiation of MSCs^6^,^7^ and agonists of β-adrenergic receptors can inhibit secretory activity of these cells^5^.

Intracellular signaling pathways linked to the activation of adrenergic receptors are well described (see refs 8, 9, 10). α1 adrenergic receptors signaling is associated mostly with G_α_ subunits of G_q/11_ class^11^,^12^ that activate phospholipase C and IP_3_ generation with Ca^2+^ release as a result. α2 adrenergic receptors are coupled with G_i_ trimeric proteins, which inhibit adenylate cyclase (AC) and do not usually induce calcium responses. However, calcium release could be caused by α2-adrenergic receptors activation through G_i_-associated G_βγ_ subunits interaction with phospholipase Cβ^13^,^14^. β-adrenergic receptors are coupled with G_s_ trimeric proteins and activate adenylate cyclase. Cyclic adenosine monophosphate (cAMP), the product of adenylate cyclase activity, activates protein kinase A (PKA) or Epac protein. β-adrenergic receptors do not potentiate Ca^2+^ signaling with the exception of some types of cardiac cells^15^.

Adrenergic receptors as well as the most of hormone receptors can undergo “desensitization”, i.e., uncoupling of receptors from their response elements, upon excessive agonist stimulation^16^. Prolonged excessive stimulation of adrenergic receptors causes the reduction of their quantities at the cell surface due to the down-regulation of receptors expression and/or elevated rate of receptor degradation^17^,^18^.

In this study, we examined the mechanisms of regulation of MSCs responsiveness to noradrenaline. Contrary to our expectations, we found that noradrenaline increases MSCs sensitivity to catecholamines in a transitory mode. We demonstrated that such increase depends on adenylate cyclase activation. The data for the first time clarify mechanisms of regulation of MSCs sensitivity to noradrenaline.

## Results

### MSCs express functionally active adrenergic receptors

**Figure 1:**
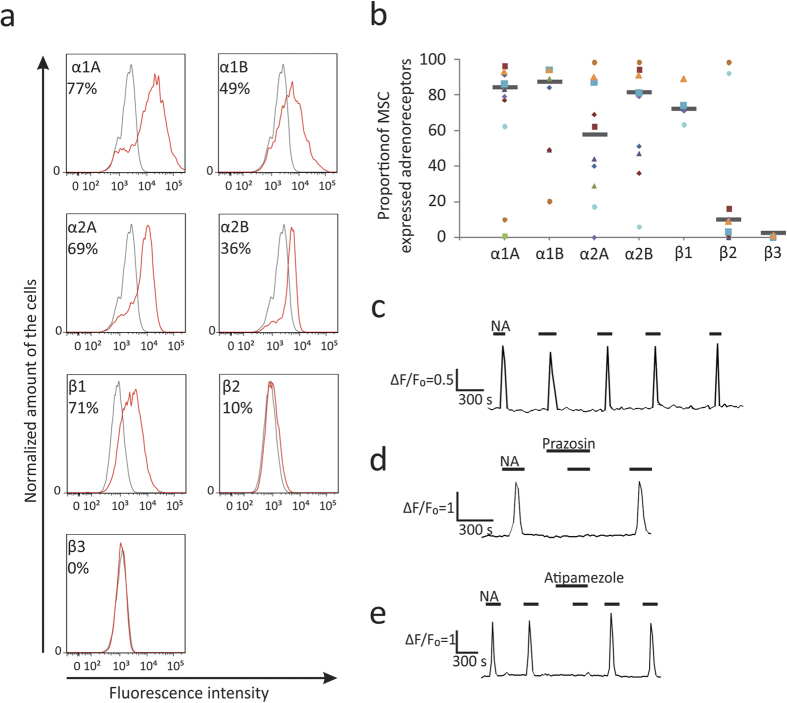
Expression of functionally active adrenergic receptors in cultured MSCs. (**a**) – Representative flow cytometry graphs. Histogram of adrenergic receptors fluorescence showed as red curve, IgG control – as gray curve. (**b**) – Proportions of cells expressing adrenergic receptors in MSCs of 10 different donors. Each symbol corresponds to one donor. Black line shows median value. (**c**–**e**) – Ca^2+^ imaging in MSCs treated with agonists of adrenergic receptors. (**c**) – Representative recordings of intracellular Ca^2+^ in individual cell, treated with noradrenaline in series (see also Supplemental Movie 2). (**d**,**e**) – Representative recordings of intracellular Ca^2+^ in individual cell, treated with noradrenaline in series in the presence of antagonists of α1- (10 μM of prazosin) and α2- (100 μM of atipamezole) adrenergic receptors. Short lines above fluorescence traces show applications of indicated compounds.

To identify adrenergic receptor isoforms expressed by MSCs we performed flow cytometry analysis using specific antibodies. MSCs expressed α1A, α1B, α2A, α2B, β1, β2 and β3 adrenergic receptors at their surface (Fig. 1a). Despite all cultures demonstrated the same immunophenotype (>99% of those cells were CD105 + CD73 + CD90 + CD45-)^19^ and similar differentiation abilities^19^, the percentage of the cells containing particular adrenergic receptor isoforms varied highly among different donors (Fig. 1b) and did not depend on donor age, sex or BMI. Overall, α-adrenergic receptors, in particular, α1A were the most abundant in MSCs. At the same time MSCs of the most donors contained low proportions of cells expressing β2 and β3 isoforms (8 out of 10 and 4 out of 4, respectively) with 6% and 1% median values. The most abundant adrenergic receptor, α1A was expressed by cells bearing MSC markers but not by possible contaminating cells, as was demonstrated by flow cytometry (Supplementary Fig. S1) and immunofluorescent staining (Supplementary Fig. S2).

**Figure 2:**
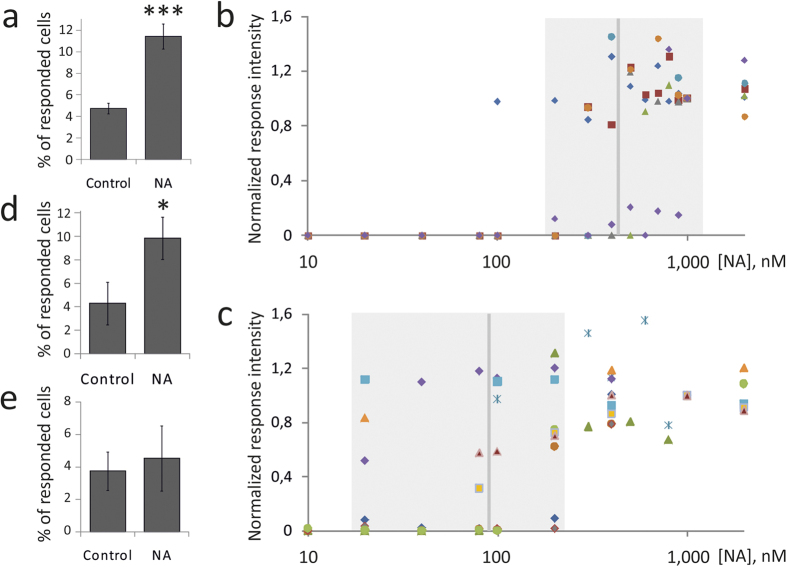
Noradrenaline increased the proportion of MSCs responsive to the hormone. (**a**) – Proportion of MSCs capable to respond to noradrenaline. NA – cells stimulated by noradrenaline for 1 hr, washed three times and incubated in full growth medium for 5 hrs followed by loading with Fluo-8 and imaging. Control - fresh growth medium was added instead of noradrenaline. Mean ± s.e.m., n = 28, ***p < 0.001 calculated with Mann-Whitney Rank Sum Test. (**b**,**c**) – Superimposed dose dependences of noradrenaline responses recorded from 8 control cells (**b**) and 13 noradrenaline treated cells (**c**). Responses of a given cell were normalized to a response induced by 1 μM noradrenaline; each symbol corresponds to an individual cell. Median values are indicated by vertical gray lines. (**d**) – Proportion of the cells responded to α1-agonist phenylephrine (100 μM) after noradrenaline treatment; n = 12, *p < 0.05 calculated with Mann-Whitney Rank Sum Test. (**e**) –Proportion of the cells responded to α2-agonist clonidine (100 μM) after noradrenaline treatment. Mean ± s.e.m., n = 10.

To ascertain whether adrenergic receptors are active we treated MSCs with noradrenaline and registered intracellular Ca^2+^ influx in individual cells. We demonstrated that cells specifically responded to serial noradrenaline applications (Fig. 1c). We expected that the most of MSCs would respond to noradrenaline by Ca^2+^ influx, since α1 were predominant adrenergic receptors expressed on the most of analyzed cells and activation of α1 adrenergic receptors triggers Ca^2+^ influx via G_q/11_/PLC/IP_3_ cascade. Surprisingly, noradrenaline induced Ca^2+^ release only in 6.9 ± 0.8% of the cells (data of 43 independent measurements) (Supplementary Movie S1). Analysis of the dose-response curves of individual MSCs has revealed that the most of responding cells demonstrate “all-or-nothing” mode with a threshold concentration 431 ± 71 nM (see Fig. 2b). The data indicate that MSCs respond to noradrenaline by activation of calcium induced calcium release (CICR), which has been demonstrated in our earlier studies^20^,^21^.

Among cells responded to noradrenaline by the activation of Ca^2+^ signaling (6.9 ± 0.8% of total population) specific α1-antagonist prazosin fully blocked noradrenaline action in 26 ± 4% of responded cells (Fig. 1d) and α2-antagonist atipamezole in 67 ± 7% of responded cells (Fig. 1e). Possibly, in the rest of cells responding to noradrenaline Ca^2+^ influx may be potentiated by α1 as well as α2-adrenergic receptors.

Therefore, despite the most of MSCs express adrenergic receptors, only small portion of them respond to noradrenaline by Ca^2+^ influx.

### Noradrenaline increases the proportion of MSCs responsive to adrenergic receptor agonists

Since hormones could regulate cell sensitivity to themselves, we hypothesized that noradrenaline may affect functional activity of adrenergic receptors in MSCs. To test this we stimulated cells with 10^−6^ M noradrenaline for 1 hour and analyzed their response to the fresh addition of this ligand 3, 6 and 24 hrs later. Noradrenaline caused more than 2-fold increase of MSCs proportion capable of responding to this hormone by the activation of Ca^2+^ signaling (Fig. 2a). However, this effect was transitory and was observed only in cells incubated for 6 hours after initial treatment with noradrenaline. Whereas cells incubated for 3 or 24 hrs responded similarly to untreated controls (Supplementary Fig. S3).

The data allowed us to suggest that noradrenaline could transiently enhance MSC sensitivity to adrenergic receptors agonists. Indeed, the threshold level of noradrenaline for CICR activation decreased up to 5 folds in MSCs preincubated with noradrenaline (from 431 ± 71 nM to 88 ± 18 nM, p < 0.001) (Fig. 2b,c). Furthermore, after noradrenaline treatment the proportion of cells responding to α1 agonist phenylephrine increased 2 times (Fig. 2d), whereas the fraction of MSCs responding to α2-adrenergic receptor agonist clonidine did not change (Fig. 2e).

**Figure 3:**
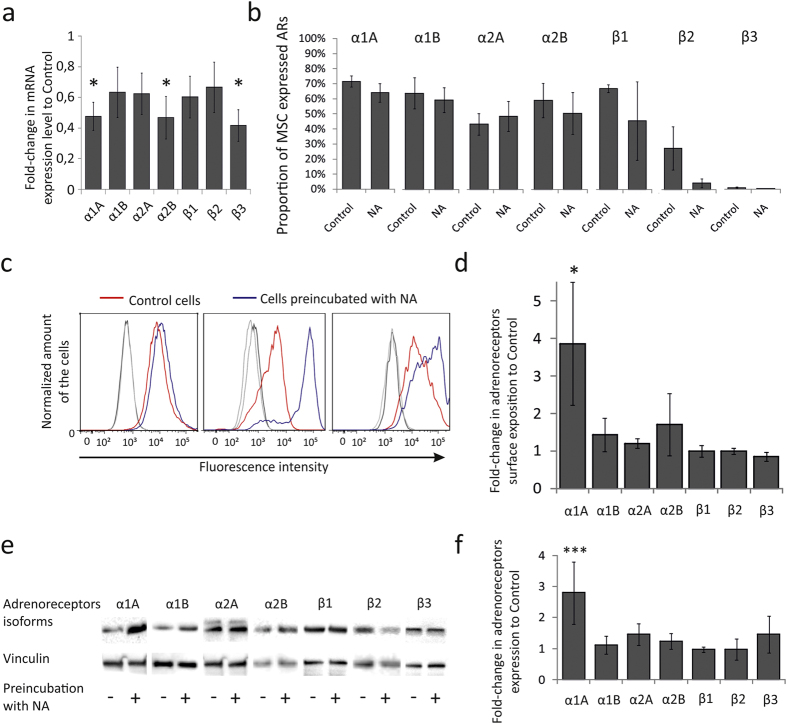
Noradrenaline treatment increased an amount of α1A-adrenergic receptors. (**a**) – mRNA of adrenergic receptors 6 hrs after noradrenaline treatment (real-time PCR). Mean ± s.e.m., n = 5, *p < 0.05 calculated with One Way ANOVA. (**b**) – Proportions of MSC expressing particular adrenergic receptors after noradrenaline treatment (NA) vs. untreated cells (Control). Mean ± s.e.m., n = 8. (**c**) – Representative flow cytometry graphs (donors 3, 4 and 8) of α1A-adrenergic receptors in MSC treated with noradrenaline (blue curves) versus cells pretreated with growth medium only (red curves). IgG controls showed as gray curves. (**d**) – The mean fold-change of the fluorescence intensity in MSCs treated with noradrenaline as compared to vehicle treated cells. Mean ± s.e.m., n = 8, *p < 0.05 calculated with Kruskal-Wallis One Way ANOVA on Ranks. (**e**) – Representative cropped Western Blot membranes of adrenergic receptors isoforms content in MSCs treated with noradrenaline or vehicle. Full-length blots are presented in Supplementary Fig. S6. (**f**) – The mean fold-change in the Western Blot adrenergic receptors bands intensity of noradrenaline pretreated cells as compared to vehicle treated cells. Mean ± s.e.m., n = 10, *p < 0.05 calculated with Kruskal-Wallis One Way ANOVA on Ranks.

To examine the mechanism of this transitory increase, we examined the expression of adrenergic receptors in MSCs 6 hrs after noradrenaline treatment. The level of mRNAs encoding α1B, α2A, β1 and β2 adrenergic receptors did not change, whereas mRNAs encoding α1A, α2B and β3 receptors have even significantly decreased, indicating that noradrenaline did not stimulate the transcription of adrenergic receptors (Fig. 3a). Proportions of MSCs expressing particular adrenergic receptors also did not change (Fig. 3b), although β1 and β2 adrenergic receptors tended to decrease. However, the mean fluorescence intensity for α1A adrenergic receptors was significantly increased by 50% in the cells preincubated with noradrenaline as compared to vehicle treated cells (Fig. 3c,d), indicating increased exposition of α1A adrenergic receptors at the cell surface. The level of other adrenergic receptor isoforms did not change. Consistently with this observation, the content of α1A adrenergic receptor was elevated in MSCs preincubated with noradrenaline (Fig. 3e,f). Inhibition of lysosomal degradation pathway by chloroquine prevented the increase of α1A adrenergic receptor on the cell surface (Supplementary Fig. S4). The data indicate that lysosomal degradation pathway and receptor recycling are involved in the elevation of α1A adrenergic receptors level on the cell surface caused by noradrenaline treatment.

Thus, noradrenaline transiently up-regulates MSC sensitivity to adrenergic receptor agonists owing to elevation of total cellular content of α1A adrenergic receptors and their exposure on the cell surface.

### Intracellular mechanisms of changing the hormonal sensitivity of MSCs

**Figure 4:**
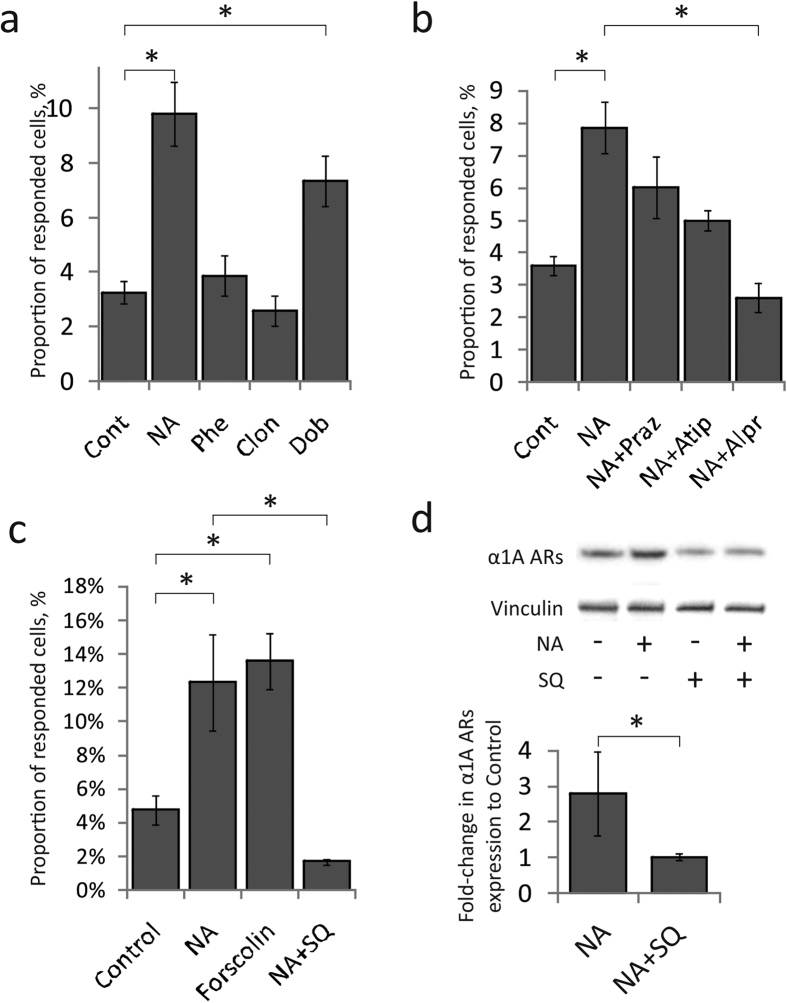
β-adrenergic receptors and adenylate cyclase regulated the number of MSC responding to noradrenaline. (**a**) – Proportion of MSCs responding to noradrenaline 6 hrs after pretreatment with adrenergic receptors agonists (1 μM of noradrenaline (NA), 100 μM of α1-agonist phenylephrine (Phe), 100 μM of α2-agonist clonidine (Clon), 1 μM of β-agonist dobutamine (Dob) or Vehicle (Cont)) instead of noradrenaline. Mean ± s.e.m., n = 14–21, *p < 0.05 calculated with Kruskal-Wallis One Way ANOVA on Ranks. (**b**) – Proportion of MSCs responding to noradrenaline 6 hrs after pretreatment with noradrenaline with adrenergic receptors antagonists (10 μM of α1-antagonist prazosin (Praz), 100 μM of α2-antagonist atipamezole (Atip), 10 μM of β-antagonist alprenolol (Alpr) or Vehicle (Cont)). Mean ± s.e.m., n = 6–12, *p < 0.05 calculated with Kruskal-Wallis One Way ANOVA on Ranks. (**c**) – Proportion of MSCs responding to noradrenaline 6 hrs after pretreatment with adenylate cyclase activator forscolin (1 μM) or by noradrenaline with 1 μM of adenylate cyclase inhibitor SQ22536 (SQ). Mean ± s.e.m., n = 3–6, *p < 0.05 calculated with Kruskal-Wallis One Way ANOVA on Ranks. (**d**) – α1A-adrenergic receptors in MSCs treated with noradrenaline (NA) or noradrenaline together with SQ22536 (SQ). Full-length blots are presented in Supplementary Fig. S6. The mean fold-change in the Western Blot bands intensity of NA pretreated cells as compared to vehicle treated cells. Mean ± s.e.m., n = 5, *p < 0.05 calculated with Mann-Whitney Rank Sum Test.

Next, we revealed the receptors and signaling cascades, which control the increase in the sensitivity of MSCs after noradrenaline treatment. To ascertain adrenergic receptors isoforms, of which activation is responsible for increased sensitivity of MSCs, we treated cells with specific agonists of adrenergic receptors or with noradrenaline mixed with specific antagonists of adrenergic receptors and 6 hours later examined their response to noradrenaline. α1 adrenergic receptors agonist phenylephrine and α2 agonist clonidine did not affect the number of responding cells. However, β adrenergic receptors agonist dobutamine increased the number of MSCs responding to noradrenaline (Fig. 4a). β adrenergic receptor antagonist alprenolol significantly reduced the number of responding cells compared to MSCs preincubated with noradrenaline (Fig. 4b). We also performed additional controls and demonstrated that dobutamine increases the ratio of cells responding to noradrenaline by activation of α1 adrenergic receptors (Supplementary Fig. S5). Thus, activation of β-adrenergic receptors is required for the increase of responding MSCs proportion.

Since β adrenergic receptors activation triggers cAMP generation by adenylate cyclase (AC), we tested if cAMP is indeed responsible for the increased MSCs sensitivity. As expected, AC activation by forskolin has increased the MSCs sensitivity to noradrenaline, whereas AC inhibitor SQ22536 decreased the number of responding cells up to the control level (Fig. 4c). Also, SQ22536 prevented the elevation of α1A adrenergic receptors cellular content caused by noradrenaline preincubation (Fig. 4d).

Taken together, noradrenaline caused transitory increase of MSCs sensitivity to catecholamines via activation of β-adrenergic receptors, adenylate cyclase and cAMP synthesis, which led to the elevation of α1A-adrenergic receptors synthesis and exposition at the cell surface.

## Discussion

This study for the first time demonstrates that noradrenaline regulates MSCs sensitivity to catecholamines. The stimulation with noradrenaline switches the adrenergic signaling from β-receptor/AC/cAMP to α1-receptors/PLC/Ca^2+^ pathways in MSCs. To our knowledge, this is the first evidence of importance of α1-adrenergic signaling in MSCs. Here, as well as in our previous work^21^, we showed that MSCs express not only functionally active β-adrenergic receptors, but also α1- and α2- isoforms. Involvement of β-adrenergic receptors in osteogenic differentiation and production of cytokines was previously demonstrated in bone marrow MSCs but precise proportion of cells expressing these receptors in whole population was not examined^5^. Physiological significance of increased α1-adrenergic signaling in MSCs remains to be solved. It has been shown, that β-adrenergic receptors decrease MSCs secretory activity^5^, whereas α1A-adrenergic receptors stimulation dramatically increase paracrine activity of cardiac cells^22^. Furthermore, calcium plays a key role in the exocytosis of paracrine factors and microvesicles^23^. Therefore, we suppose that adrenergic signaling switch from β-receptor/AC/cAMP to α1-receptors/PLC/Ca^2+^ could lead to increased secretory activity of these cells which is critically important for MSC’s functional activity^19^.

Exact mechanism, by which β-adrenergic receptors/AC/cAMP pathway regulates the level of α1-adrenergic receptors, requires further experimentation. Here we demonstrated that noradrenaline does not up-regulate α1A-adrenergic receptors gene transcription. That may be due to either translation activation or suppression of receptor’s intracellular degradation. cAMP/PKA signaling module may regulate both protein translation and degradation, however, the activation of such signaling usually results in a decrease of protein content^24^,^25^,^26^,^27^,^28^. Other possible way of protein level control is the regulation of adrenergic receptors-specific microRNAs expressed in MSCs, such as miR-181a,b and miR-132^29^,^30^. Using chloroqiune, which blocks lysosomal degradation and facilitates the accumulation of recycling proteins in the endosomal compartment^31^, we demonstrated that noradrenaline may enhance α1A-adrenergic receptors recycling. Importantly, chloroqiune treatment only prevented the appearance of additional α1A-adrenergic receptors on the cell surface. This might indicate that cells have different receptor pools - constitutive (slowly recycling) and inducible (fast recycling) ones. Let us speculate that slowly recycling receptors have low sensitivity to basal concentrations of noradrenaline (10^−6^ M), then this may explain why the majority of receptor expressing cells do not respond to noradrenalin by Ca^2+^ influx.

We demonstrated in this study that only a small portion of MSCs expressing α1- and α2- adrenergic receptors responds to noradrenaline by intracellular calcium release. Since we did not investigate the activation of alternative intracellular pathways, such as MAP kinase –dependent ones^22^, we cannot conclude that the majority of α1-adrenergic receptors are completely “silent” in MSCs, which did not respond to noradrenaline by intracellular calcium release. However, our data indicate that noradrenaline causes not only MSCs sensitization to catecholamines but also a “maturation” of α1-receptors/PLC/Ca^2+^ pathway in these cells. This looks similar to the maturation of adrenergic signaling during embryogenesis. There, β-adrenergic receptors arise much more earlier in course of embryonic development than catecholamines begin to be synthesized and sympathetic cells are formed^32^. β-adrenergic receptors of heart cells are not desensitized nor down-regulated by β-agonists during embryonic and neonatal development^33^. Moreover, the stimulation by β-agonist leads to sensitization and increased responsiveness of target cells. β-adrenergic receptors stimulation leads to both increase in the receptors density and changes in the level of G-proteins with specific shift in signaling to favor G_s_ protein as compared to G_i_ and changes in AC isoforms^18^. Here, we also demonstrate that noradrenaline led to transitory sensitization rather than desensitization of MSCs to catecholamines. We showed, that in contrast to embryonic tissues AC activation in MSCs led to specific rising in α1A adrenergic receptors expression level and subsequent increase in both sensitivity and the number of responded cells. Task of the next studies may be the question whether noradrenaline changes the level of G-proteins and/or AC isoforms.

Taken together, our data indicate that MSCs preserve embryonic adrenergic signaling regulation. Considering that noradrenaline exerts evident effects to MSCs secretory activity, these data are important for understanding their physiology and regulation of functional activity.

## Methods

### MSCs isolation and culturing

MSCs were isolated from subcutaneous fat tissue of 14 healthy young donors (age 45,25 ± 4,11 years; BMI 23.4 ± 2.5) using enzymatic digestion as previously described^34^. All experiments were carried out in accordance with approved guidelines. All donors gave their informed consent and the local ethics committee of city clinical hospital #31 (Moscow, Russia) approved the study protocol. Cells were cultured in AdvanceSTEM Mesenchymal Stem Cell Media containing 10% AdvanceSTEM Supplement (HyClone), 1% antibiotic–antimycotic solution (HyClone) at 37 °C in 5% CO_2_ incubator. Cells were passaged at 70% confluency using HyQTase solution (HyClone). For the experiments, MSCs cultured up to 3rd–4th passages were used. To confirm their multipotency, MSCs were induced into osteogenic, adipogenic and chondrogenic differentiation as described earlier^35^.

### Flow cytometry

MSC immunophenotype and the proportion of cells expressing adrenergic receptors were analysed using flow cytometry. After medium harvesting cells were detached from culture dishes using Versen solution and stained with antibodies against CD45 (BD Pharmingen, № 557748), CD73 (BD Pharmingen, № 550257), CD90 (BD Pharmingen, № 555597) and CD105 (BD Pharmingen, № 560819) as described elsewhere. Experiments of coexpression adrenergic receptors and MSC markers were provided with MSC Phenotyping Kit human (Miltenyi Biotec Inc, USA). Adrenergic receptors were detected using specific antibodies α1A (abcam ab137123, dilution 1:250), α1B (abcam ab169523, 1:100), α2A (abcam ab65833, 1:100), α2B (abcam ab151727, 1:100), β1 (abcam ab3442, 1:100), β2 (abcam ab61778, 1:100), β3 (Acris, H00000155-B01P, 1:100), following by secondary antibodies AlexaFluor 647 (Jackson Immunoresearch, 711-606-152,1:500) and AlexaFluor 594 (Jackson Immunoresearch, 111-586-045, 1:500) or DyLight549-conjugate Affinipure Goat AntiMouse IgG (H + L) (Jackson Immunoresearch, 115-505-146) on formalin fixed non-permeabilized cells. Normal rabbit IgG (10500C, Invitrogen, 1:300), normal mouse IgG1 (Dako X0931, 1:20), PE-Cy5 mouse IgG1 Isotype Control (560819, BD, 1:100) and mouse IgG1/RPE Isotype Reagent (X0928, Dako, 1:25) were used as a negative control. Stained cells were analyzed using flow cytometry scanner BDLSR Fortessa Special Order Research Product (BD Pharmingen, USA). 20,000–40,000 events were acquired and analyzed for antigen expression. We evaluated the proportion of cells expressing particular adrenergic receptors on 2D flow cytometry plots using FlowJo software. Changes in receptor expression level were determined as a difference between the fluorescence intensities in control and noradrenaline treated cells. Only those cells which expressed particular receptors were taken for analysis of fluorescence intensities.

### MSCs treatment and Ca^2+^ imaging

Adrenergic receptor activation was assessed using Ca^2+^ imaging in individual cells. Briefly, cells were seeded in 24-well plate at low density to prevent cell-to-cell communications during the imaging. MSCs were loaded with Fluo-8 (abcam, ab142773), 4 μM in Hanks Balanced Salt Solution with 20 mM Hepes, for 1 hour. To stimulate Ca^2+^ transients in serial mode MSCs were treated by noradrenaline (Calbiochem, Cat# 489350, 1 μM) alone or together with α1-adrenergic receptor antagonist prazosin (Abcam, ab120238, 10 μM) or α2-adrenergic receptor antagonist atipamezole (Abcam, ab120785, 100 μM). To evaluate the input of particular adrenergic receptor in the activation of Ca^2+^ transients we treated cells with receptor antagonists, 5 min before noradrenaline addition. Cells were stimulated with noradrenaline for 3 min, and then washed by Hanks solution 3–5 times for 5 min following by the next stimulation with noradrenaline. Up to 8 additions of ligands were used. Threshold concentration was determined using addition of 0.01 – 10 μM noradrenaline in series. To analyze the effect of noradrenaline on MSCs responsiveness, cells were stimulated by noradrenaline for 1 hr, washed three times and incubated in full growth medium for 2, 5 or 23 hrs followed by loading with Fluo-8 for 1 hr. To evaluate what isoforms of adrenergic receptors are involved in this noradrenaline effect, we stimulated MSCs with either α1-agonist phenylephrine (Abcam, ab120761, 100 μM), α2-adrenergic receptor agonist clonidine (Abcam, ab120753, 100 μM), β1/β2-agonist dobutamine (Abcam, ab120768, 1 μM) or antagonists of adrenergic receptors (prazosin, atipamezole or β-antagonist alprenolol (Sigma, A8676, 10 μM)). To evaluate the input of adenylate cyclase activation in noradrenaline effect we stimulated cells with adenylate cyclase activator forscolin (Abcam, ab120058, 1 μM) or noradrenaline mixed with non-competitive adenylate cyclase inhibitor SQ22536 (Abcam, ab120642, 1 μM) before imaging. To measure the percent of responding cells we recorded the baseline for 5 min then once added noradrenaline, phenylephrine or clonidine. Ca^2+^ transients were measured in individual cells using inverted fluorescent microscope Nikon Eclipse Ti equipped with an objective CFI Plan Fluor DLL 10X/0.3 (Nikon) and with digital EMCCD camera Andor iXon 897 (Andor Technology). We used the simultaneous measuring of 36 or 42 fields of view in Large Image mode to increase the number of analyzed cells. Movies were analyzed using NIS-Elements (Nikon) and ImageJ software. Alterations of cytosolic Ca^2+^ from the resting level were quantified as a difference of Fluo-8 fluorescence intensity (ΔF/F0) recorded from an individual cell. The percent of responding cells was measured as ratio of responded cells to all cells in the field of view.

### RNA Isolation and Quantitative RT-PCR

Total cellular RNA was isolated from cultured MSCs using Direct-Zol RNA Mini Prep (Zymo Research, R2025) according to manufacturer instructions. To determine the expression of adrenergic receptors cDNA was synthesized using Super Script III Reverse Transcriptase (Fermentas, P/N 56575) with oligo-dT (Invitrogen, P/N 58063) DTT 0,1M (Invitrogen, P/N Y00147), RiboLok RNase inhibitor (Fermentas, #EO0381) according to manufacturer’s instruction. Real-time PCR was performed using ready-to-use reaction mix, containing DNA polymerase, SYBR Green and ROX (Evrogen) in 7500 Fast Real-time PCR system (Applied Biosystems). Following oligonucleotide primers were used for amplification:

Fold change of mRNA expression was calculated using 2^−ΔΔCt^ method, RPL-13A was used as a reference gene. The following primers were used for Quantitative RT-PCR: Gene ADRA1A, 189 bp, forward AGAAGAAAGCGGCCAAAACG, reverse TGGAGCATGGGTATATGATGGG; Gene ADRA1B, 191 bp, forward CCACAACACATCAGCACCTG, reverse AGGCCACAGACAAGATGACTAG; Gene ADRA2A, 195 bp, forward CCCCTTCTTCTTCACCTACACG, reverse AAACCTCACACGATCCGCTTC; Gene ADRA2B, 193 bp, forward ATCCCCGATCACTGGCATTC, reverse AGGAAGACAGGTGGTGGTAAAC; Gene ADRB1, 190 bp, forward AGGAAAGTTTGGGAAGGGATGG, reverse CAGAGAGTGTCAAAAACCACCTG; Gene ADRB2, 195 bp, forward ATGCCAATGAGACCTGCTGT, reverse GCTCCACCTGGCTAAGGTTC; Gene ADRB3, 165 bp, forward CAGTCCCTGCCTATGTTTG, reverse TTCCTGGATTCCTGCTCT.

### Western-blotting

Cells were grown to 60% confluence in 100 mm Petri dishes with DMEM containing 10% FBS. SQ22536 or vehicle, if necessary, were added 30 min prior to stimulation with noradrenaline. The cells stimulated with 10^−6^ M noradrenaline, incubated for 1 hour and washed for three times with growth medium. 6 hours after stimulation cell medium was quickly removed and the plates were transferred on ice. The cells were rinsed with an ice-cold phosphate buffered saline (5.2 mM Na_2_HPO_4_, pH 7.4 and 150 mM NaCl) and scraped by rubber cell scraper (Corning) in 3× SDS sample buffer (15% β-mercaptoethanol, 6% SDS, 0.2 M Tris, pH 6.8, 40% glycerol and 0.03% bromophenol blue). Then samples wereboiled for 4 min, cooled on ice and passed 5 times through a 30-gauge needle to splinter DNA.

Proteins were separated by AnykD Mini-PROTEAN TGX Stain-Free Protein Gels (BioRad, USA) in the Mini-PROTEAN 3 BioRad Units using Tris/Glycine/SDS buffer (diluted from 10× premixed electrophoresis buffer, contained 25 mM Tris, 192 mM glycine, 0.1% SDS, pH 8.3, BioRad). The loading amount was controlled by immunostaining for vinculin. The proteins were transferred onto 0.45 μm polyvinylidene fluoride (PVDF) membranes (Amersham, USA) for 1 h at 350 mA in the buffer containing 25 mM Tris, 0.192 M glycine, pH 8.3, 20% ethanol, 0.02% sodium lauryl sulfate. The membranes were blocked for 1 hour in 5% non-fat milk in TBST (25 mM Tris/HCl, pH 7.4, 150 mM NaCl, 0.1% Tween-20). The membranes were incubated overnight at 4 °C with an appropriate primary antibody in a dilution recommended by the supplier, washed 3 times and incubated for 1 hour at the room temperature with the horseradish peroxidase conjugated secondary antibodies. All blocking procedures, antibody incubation and washings were carried out in TBST (25 mM Tris/HCl, pH 7.4, 150 mM NaCl, 0.1% Tween-20) supplemented with 5% dry nonfat milk. The protein bands were visualized by enhanced chemiluminescence (ECL, West Pico, Pierce, USA) on ChemiDoc Imaging System (BioRad, USA). Each membrane was developed for at least two different time intervals to ensure the linearity of the ECL signal. The quantitative analysis was achieved using ChemiDoc Imaging System (BioRad, USA) software.

### Statistics

Statistical analysis was performed using SigmaPlot 11.0 software. Data was assessed for normality of distribution using the Kolmogorov-Smirnov test. Values are expressed as mean  ±  standard error of mean (s.e.m.). Comparison of two independent groups was performed by Student t-test for normally distributed data and Mann–Whitney U-criteria (M-U test) for not normally distributed data. Multiple comparisons were made using Kruskall-Wallis test with subsequent application of Dann criteria. Statistical significance was defined as p-value < 0.05.

## Additional Information

**How to cite this article**: Tyurin-Kuzmin, P. A. *et al*. Activation of β-adrenergic receptors is required for elevated α1A-adrenoreceptors expression and signaling in mesenchymal stromal cells. *Sci. Rep.* **6**, 32835; doi: 10.1038/srep32835 (2016).

## Supplementary Material

Supplementary Movie 1

Supplementary Information
