# Supplementary material for: Activation of β-adrenergic receptors is required for elevated α1A-adrenoreceptors expression and signaling in mesenchymal stromal cells
Source: Sci Rep. 2016 Sep 6;6:32835. doi: 10.1038/srep32835 (PMC5011778; doi:10.1038/srep32835)

Supplementary information

Activation of β-adrenergic receptors is required for elevated α1A-adrenoreceptors expression and signaling in mesenchymal stromal cells

Tyurin-Kuzmin Pyotr A.[#]*, Fadeeva Julia I.[#], Kanareikina Margarita A., Kalinina Natalia I., Sysoeva Veronika Yu., Dyikanov Daniyar T., Stambolsky Dmitriy V., Tkachuk Vsevolod A.

Department of Biochemistry and Molecular Medicine, Faculty of Fundamental Medicine, M.V. Lomonosov Moscow State University, Moscow, Russia

Supplementary Movie S1. Responses of MSCs on repetitive noradrenaline adding. NA indicates time intervals when 1μM noradrenaline was added.

Supplementary Figure S1. Representative flow cytometry dot plot graphs of MSCs stained with antibodies against α1A adrenergic receptors and MSC markers. IgG controls are showed by gray dots.

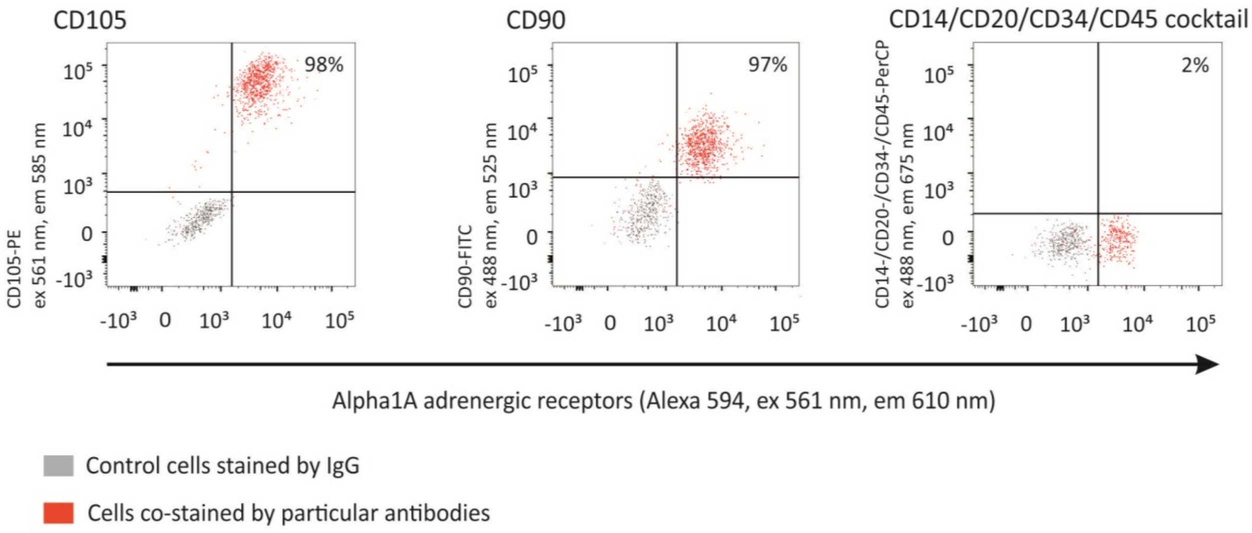

Supplementary Figure S2. α1A adrenergic receptors and MSC markers co-expression in MSCs. α1A adrenergic receptors – red fluorescence; MSC markers (CD90 and CD105) – green fluorescence.

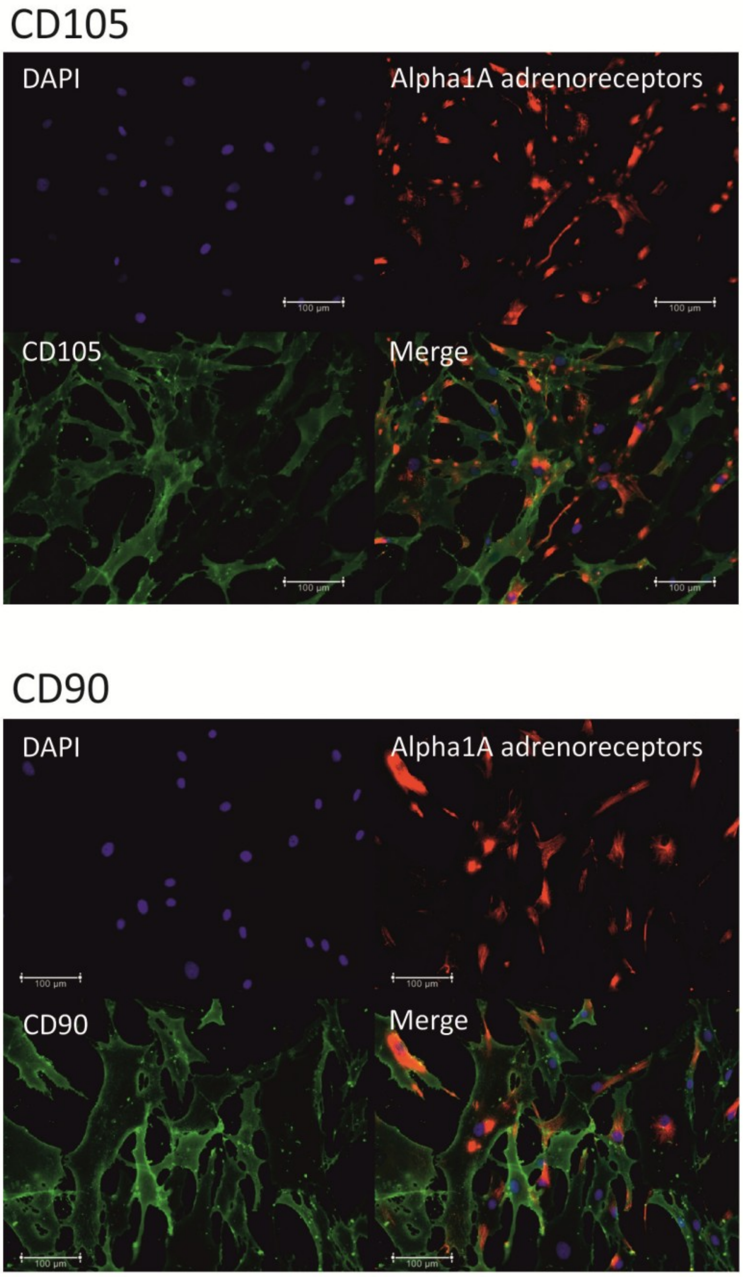

Supplementary Figure S3. Noradrenaline transiently increases the proportion of MSCs responsive to the hormone. Mean ± s.e.m., n=7-28, *p<0.05 calculated with Kruskal-Wallis One Way ANOVA on Ranks.

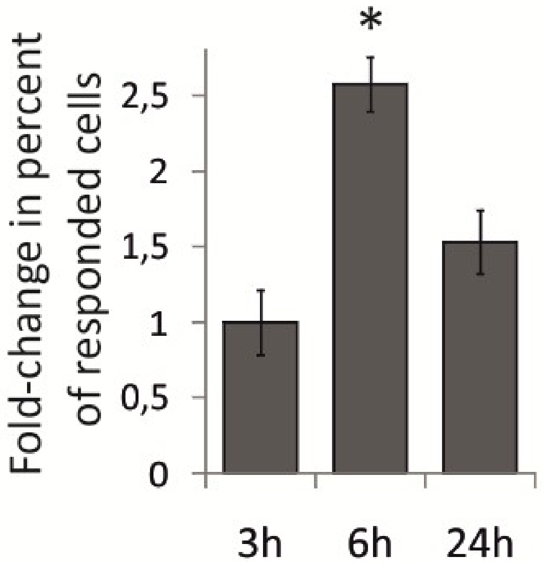

Supplementary Figure S4. Flow cytometry graphs of α1A adrenergic receptors in MSC treated with noradrenaline (violet curve) and control cells (red curve). α1A adrenergic receptors in MSCs treated by noradrenalinein the presence of lysosome inhibitor chloroquine (blue curve) and control cells incubated with chloroquine (orange curve). Typical IgG control showed as gray curve.

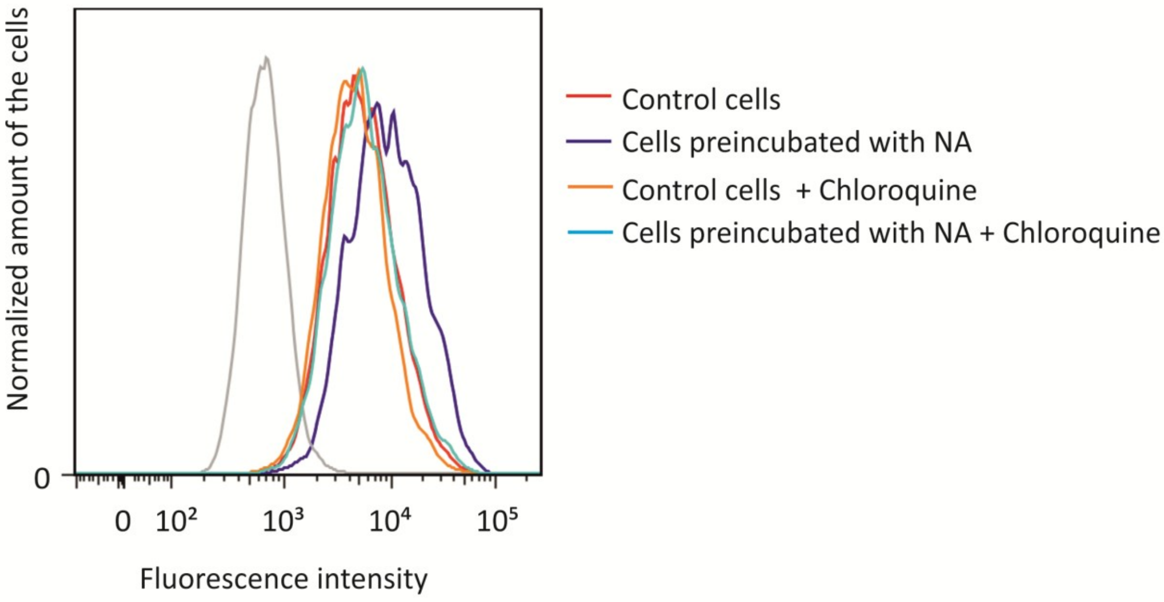

Supplementary Figure S5. β adrenergic agonist dobutamine increases the proportion of MSCs responsive to noradrenaline through α1 adrenergic receptors. The proportion of MSCs capable to respond on noradrenaline was analyzed 5 hrs after treating cells with dobutamine for 1 hr. Ca^{2+} influxes were measured in the presence (NA+Praz) or absence (NA) of α1 antagonist prazosin. Responses of dobutamine treated cells were compared to vehicle treated cells and plotted as mean fold-changes of responsive MSC proportion.

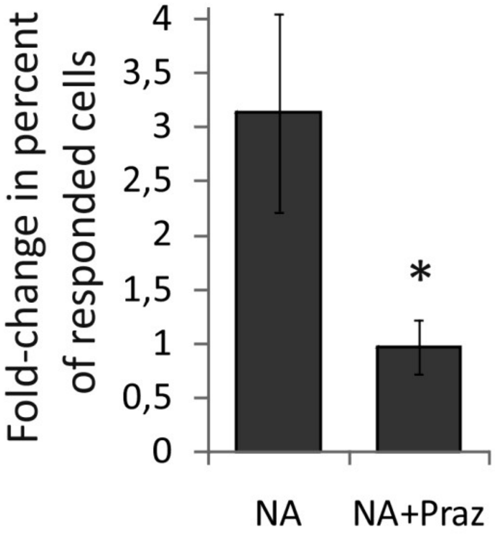

Supplementary Figure S6. Full-length Western blots for Figures 3E and 4D.

Full-length Western blots to Fig. 3E

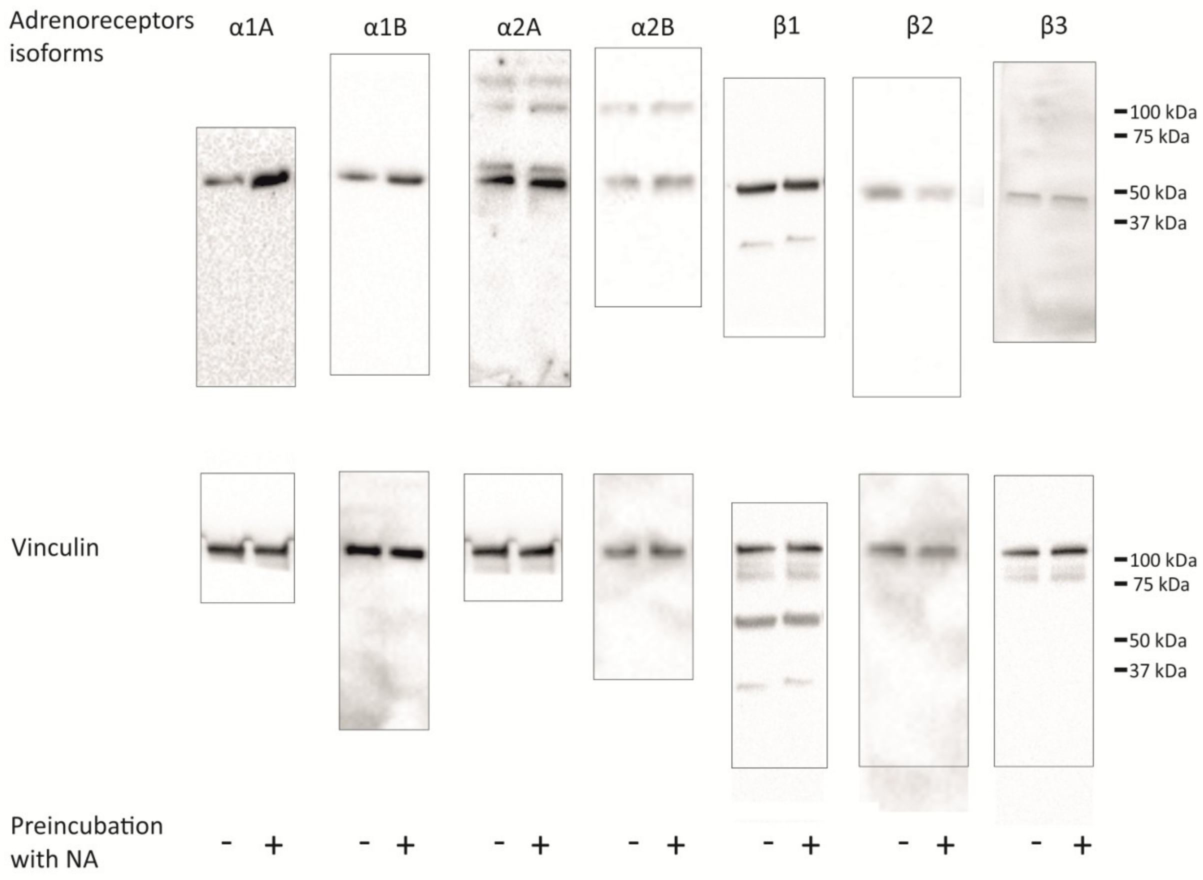

Full-length Western blots to Fig. 4D

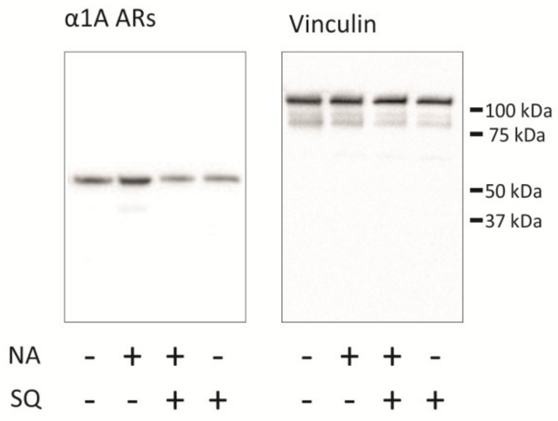

Supplement: Supplementary Information [file srep32835-s2.pdf]
